# Mitogen- and Stress-Activated Protein Kinases 1 and 2 Are Required for Maximal Trefoil Factor 1 Induction

**DOI:** 10.1371/journal.pone.0063189

**Published:** 2013-05-13

**Authors:** Protiti Khan, Bojan Drobic, Beatriz Pérez-Cadahía, Shannon Healy, Shihua He, James R. Davie

**Affiliations:** Manitoba Institute of Child Health, University of Manitoba, Winnipeg, Manitoba, Canada; Albany Medical College, United States of America

## Abstract

Mitogen- and stress-activated protein kinases 1 and 2 (MSK1 and MSK2), activated downstream of the ERK- and p38-mitogen-activated protein kinase pathways are involved in cell survival, proliferation and differentiation. Following mitogenic or stress stimuli, they mediate the nucleosomal response, which includes phosphorylation of histone H3 at serine 10 (H3S10ph) coupled with transcriptional activation of immediate-early genes. While MSK1 and MSK2 are closely related, their relative roles may vary with cellular context and/or stimuli. However, our knowledge of MSK2 recruitment to immediate-early genes is limited, as research has primarily focused on MSK1. Here, we demonstrate that both MSK1 and MSK2, regulate the phorbol ester 12-O-tetradecanoylphorbol-13-acetate induced expression of the breast cancer marker gene, trefoil factor 1 (*TFF1*), by phosphorylating H3S10 at its 5′ regulatory regions. The MSK-mediated phosphorylation of H3S10 promotes the recruitment of 14-3-3 isoforms and BRG1, the ATPase subunit of the BAF/PBAF remodeling complex, to the enhancer and upstream promoter elements of *TFF1*. The recruited chromatin remodeling activity leads to the RNA polymerase II carboxy-terminal domain phosphorylation at the *TFF1* promoter, initiating *TFF1* expression in MCF-7 breast cancer cells. Moreover, we show that MSK1 or MSK2 is recruited to *TFF1* regulatory regions, but as components of different multiprotein complexes.

## Introduction

Mitogen- and stress-activated protein kinases 1 and 2 (MSK1 and MSK2) are activated downstream of the ERK- and p38-mitogen-activated protein kinase (MAPK) signal transduction pathways, following exposure to various stimulants or stresses including cytokines, growth factors, pharmacological mitogens or UV irradiation. They are involved in cell survival, proliferation and differentiation [1]–[3]. The crucial role that MSKs play in tumor formation was demonstrated in a study showing that MSK1/2 knockout mice developed significantly fewer skin tumors than wild-type mice in a two-stage chemical carcinogenesis model [4]. Moreover, it was demonstrated that when MSK1 was knocked down in the metastatic Hras1-transformed murine fibroblast cell line Ciras-3, these cells lost their capability for anchorage-independent growth, thereby losing their malignant phenotype [5].

Once activated, MSKs phosphorylate downstream targets, including transcription factors and chromatin substrates. Phosphorylation of the NH_2_-terminal tail of histone H3 at serine 10 or serine 28 and phosphorylation of nucleosome-binding protein HMGN1 at serine 6 are among the downstream events mediated by MSKs and have been collectively termed the ‘nucleosomal response’ [6]. This H3 phosphorylation event is rapidly inducible and is associated with transcriptional activation of immediate-early (IE) genes, such as *Jun*, *Fos*, *Fosl1* and *Cox-2* [7], [8]. H3S10ph and H3S28ph are implicated in upstream promoter chromatin remodeling that is required for initiation of transcription [7], [9]. However, it has yet to be shown if the nucleosomal response is involved in enhancer remodeling.

The MSKs share 61.5% identity in their amino acid sequences (Fig. S1), and there is evidence that they do not have redundant functions. For example, in primary embryonic fibroblasts exposed to mitogenic and stress stimuli MSK1 and MSK2 both contributed to CREB activation and the nucleosomal response. However, the nucleosomal response was more impacted in MSK2 than in MSK1 knockout fibroblasts [6], [10]. MSK2, but not MSK1 was also shown to be critical for UV-induced phosphorylation of NFκB p65 in MDA-MB-231 cells [11]. In contrast, cocaine-stimulated H3 phosphorylation and IE gene induction were dependent on MSK1 in striatal neurons [12]. Additionally, MSK1 and MSK2 expression profiles in various normal and cancer tissues and cell lines appeared to differ between each other [13]. Another study indicated that murine primary embryonic fibroblasts had higher levels of MSK2 than embryonic stem cells, where MSK2 could not be detected [10]. However, most studies either focused on MSK1 or did not distinguish between MSK1 and MSK2 as they used double MSK1/MSK2 knockout mice [14]–[18]. Thus, our knowledge of MSK2 recruitment to IE genes is lacking.

In the cases of MSK1- and MSK2-co-mediated responses, it is currently unknown whether MSK1 and MSK2 act together or separately to induce the expression of IE genes, whether they target the same genes or the same alleles, or if they are components of the same complexes. To address these questions, we investigated the mitogenic induction of the trefoil factor 1 (*TFF1*) gene by the phorbol ester 12-O-tetradecanoylphorbol-13-acetate (TPA). *TFF1*, encoding a secretory protein with proinvasive and angiogenic effects, has an elevated expression in estrogen receptor α positive (ER+) breast cancers [19]–[21]. TFF1 protein is a marker for ER+, well-differentiated, low-grade breast cancers (about 65% of all breast cancers) [22], [23]. The *TFF1* gene is regulated by an enhancer located 10.5 kb upstream of the transcription initiation site [24]. Transcription factors and coactivators associated with the enhancer and upstream promoter element (UPE) are involved in the transcriptional activation of the *TFF1* gene [25], [26]. Both estrogen and phorbol esters (e.g. TPA) can mediate the expression of the *TFF1* gene, albeit via different mechanisms [27]. The chromatin remodeling events taking place at the *TFF1* UPE have been extensively studied in estrogen-driven induction of *TFF1* [28]–[30], whereas phorbol ester induction of *TFF1* is not as well understood. Previously, we reported that TPA addition to MCF-7 breast cancer cells cultured under estrogen-free serum starved conditions resulted in the recruitment of MSK1 to the *TFF1* UPE, along with increased H3S10ph (but not H3S28ph) and H3 acetylation (H3K9acK14ac) levels [27]. However, the recruitment of MSK2 or the mechanistic consequences of H3S10 phosphorylation in the context of the TPA-induced *TFF1* transcriptional activation have not been explored. Recruitment of MSK1 and/or MSK2 at the TFF1 enhancer region was not examined.

Here, we show that MSK1 and MSK2 belong to different multiprotein complexes but both mediate chromatin remodeling that is required at the enhancer and UPE for TPA-induced initiation of *TFF1* expression in MCF-7 breast cancer epithelial cells.

## Materials and Methods

### Cell Culture Conditions

Human breast cancer cell lines MCF-7 and ZR75 (ATCC) were grown in Dulbecco’s modified Eagle’s medium (DMEM) supplemented with 10% fetal bovine serum, penicillin (100 units/ml), streptomycin (100 mg/ml), and 0.3% glucose in 37°C humidified incubator with 5% CO_2_. MCF-7 cells were grown up to 85% confluence and then cultured in phenol red-free DMEM supplemented with penicillin (100 units/ml), streptomycin (100 mg/ml), 0.3% glucose, 0.1% (v/v) bovine serum albumin and apo-transferrin (10 µg/ml) for 72 h in order to drive the cell population into G_0_-G_1_. Cells were either untreated or treated with 100 nM TPA for 15, 30, 45 and 60 min. In inhibition studies, cells were pretreated with MAPK inhibitor UO126 (10 µM), or MSK inhibitor H89 (10 µM) for 30 min alone or followed by treatment with TPA.

### Chromatin Immunoprecipitation (ChIP) and re-ChIP Assays

**Figure 2:**
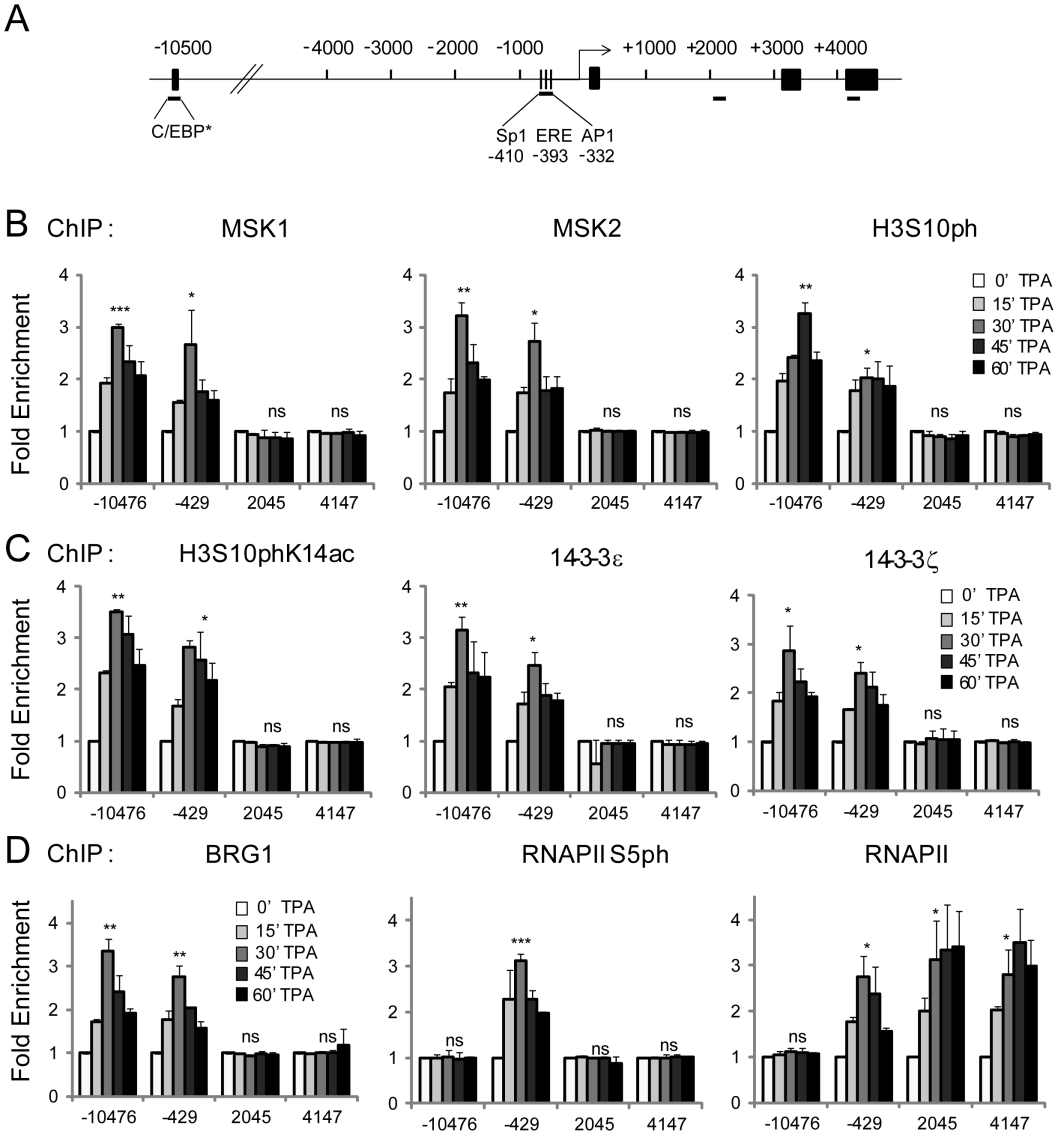
Distribution of MSK1/2, H3S10ph, H3S10phK14ac, 14-3-3ε/ζ, BRG1, RNAPolIIS5ph, and RNAPolII at regulatory and coding regions of *TFF1* in response to TPA. A. Schematic representation of *TFF1* gene structure. Black bars underneath the map show regions amplified in the ChIP assay. Each region is labeled according to the 5′ position of the forward primer relative to the transcription start site. The exons are represented by black boxes, while the binding sites of relevant transcription factors located in the amplified enhancer (−10476) and UPE (−429) regions are displayed. C/EBP, CCAAT-enhancer binding protein; Sp1, GC box that is a binding site for the Sp family of transcription factors; ERE, estrogen response element; AP1 constitutes a combination of dimers formed of members of the JUN, FOS and ATF families of transcription factors. Asterisk indicates a putative binding site. B. ChIP assays were performed using antibodies against MSK1, MSK2 or H3S10ph on formaldehyde-crosslinked mononucleosomes prepared from serum-starved MCF-7 cells either untreated (0′ TPA) or treated with TPA for 15, 30, 45 and 60 min. Equal amounts of input and immunoprecipitated DNA were quantified by real-time quantitative PCR. Enrichment values are the mean of three independent experiments, and the error bars represent the standard deviation. C. ChIP experiments were performed as in B, using antibodies against H3S10phK14ac, 14-3-3ε or 14-3-3ζ. D. ChIP experiments were performed as in B and C, using antibodies against BRG1, RNAPII S5ph or total RNAPII. *P≤0.05, **P≤0.01, ***P≤0.001, ns = P≥0.05 (Student’s paired t-test).

Following TPA treatment, MCF-7 cells were crosslinked with 1% formaldehyde for 10 min at room temperature. After harvesting, the cells were resuspended in cell lysis buffer [7]. After 10 min rotation at 4°C the cellular material was spun at 2000×g for 10 min to obtain the nuclei. The nuclear pellet was resuspended in micrococcal nuclease (MNase) digestion buffer (10 mM Tris–HCl pH 7.5, 0.25 M sucrose, 75 mM NaCl, plus phosphatase/protease inhibitors) and A_260_ was measured. In order to obtain ∼150 bp DNA fragments, 2.5U of MNase per A_260_ of nuclear suspension was added in the presence of 3 mM CaCl_2_ and incubated at 37°C for 20 min. The MNase reaction was stopped by the addition of EDTA pH 8.0 (5 mM final concentration). In order to release nuclear material, the samples were adjusted to 0.5% SDS and rotated for 1 h at room temperature. Insoluble material was pelleted at 2000×g for 10 min and the soluble material was diluted to 0.1% SDS with radio immunoprecipitation assay (RIPA) buffer along with phosphatase/protease inhibitors. Twelve A_260_ of protein G-Sepharose (Pierce) pre-cleared MCF-7 lysate was incubated with 12 µl of anti-MSK1 (Sigma-Aldrich, M5437) or anti-MSK2 (Invitrogen, 38-5300), anti-H3S10ph (Santa Cruz, sc-8656-R), anti-H3S10phK14ac (Milipore, 07–081), anti-14-3-3ε (Santa Cruz, sc-1020), anti-14-3-3ζ (Santa Cruz, sc-1019), anti-BRG1 (Milipore, 07–478), anti-RNAPII S5ph (AbCam, ab5131-50) or anti-RNAPII (Santa Cruz, sc-899) overnight at 4°C. Magnetic protein G Dynabeads (Invitrogen) were added for 2 h at 4°C. For re-ChIP assays, after the elution of first ChIP, the samples were diluted 10 times with dilution buffer (15 mM Tris–HCl pH 8.1, 1% Triton X-100, 1 mM EDTA, 150 mM NaCl) and subjected to the ChIP procedure again. Negative control included performing ChIP/re-ChIP assays without adding antibody. DNA–protein fragments were processed as previously described [31], [32]. Input and ChIP/re-ChIP DNAs were quantified using PicoGreen assay. Equal amounts of input, ChIP (0.1 ng) or re-ChIP (0.05 ng) DNA were used to perform SYBR Green real time PCR on iCycler IQ5 (BioRad). The enrichment was calculated as follows: Fold enrichment = R^(Ct input – Ct ChIP)^, where R is the rate of amplification. Values for each time point were normalized to time 0 values. Primer sequences targeted the regions indicated in Fig. 2A. Primers were designed using Oligo5 software and are available upon request. Statistical analysis was performed using 2-tailed paired Student’s t-test.

### Immunoprecipitation and Immunoblotting

MCF-7 cells were harvested and lysed by sonication in cold lysis buffer (150 mM NaCl, 50 mM Tris-HCl pH 8.0, 0.5% NP-40, protease and phosphatase inhibitors). Cell-free lysate was collected by centrifugation (11950×g, 10 min, 4°C), and protein concentration was determined using the Bradford assay (Pierce). Five hundred micrograms of total cell extract were mixed overnight with 4 µg anti-MSK1, 8 µg anti-MSK2 or 4 µg control IgG (pre-immune rabbit) antibodies. Forty microliters of protein A/G sepharose beads were added the next day and mixed for 2 h at 4°C. One tenth of unbound material was set aside for PAGE analysis. Beads were washed three times with cold lysis buffer by centrifugation (2000×g, 5 min, 4°C), and eluted by incubation in SDS-PAGE buffer (15 min/92°C). Input (50 µg), unbound, bound, and nonspecific (rabbit IgG IP) samples were resolved on 8%-SDS PAGE and immunoblotted with anti-MSK1 (1 h at room temperature, 1∶8000 dilution in 5% milk in TBST) or anti-MSK2 (overnight at 4°C, 1∶500 dilution in 5% milk in TBST) antibodies. Immunolabeled bands were detected using Western-Lightning Plus-ECL Reagent (Perkin Elmer).

### RNA Isolation and Real Time RT-PCR Analysis

Total RNA from untreated and treated MCF-7 cells was isolated using RNeasy Mini Kit (QIAGEN). RNA (0.4 or 0.8 µg) was used for cDNA conversion (Invitrogen). Real-time PCR reactions were performed on iCycler IQ5 (BioRad) using SYBR Green for labeling. Fold change in RNA levels was normalized to cyclophilin33 (*CYP33*) or GAPDH levels in untreated and treated samples. Primer sequences were designed using Oligo5 software and are available upon request. Primers targeted exon 3 of *TFF1* and exon 2 of *CYP33*.

### Generation of Transient MSK1 and MSK2 Knockdown MCF-7 Cells

MCF-7 cells were seeded at 2.5×10^5^ cells per well in 6-well plates and allowed to adhere for 24 h. The medium was replaced with antibiotic-free DMEM containing 5% FBS and the cells were left for 1 h. Cells were then transfected with 50 nM scramble (Non-targeting Pool), human MSK1 (RPS6KA5, NM_182398) or MSK2 (RPS6KA4, NM_001006944) ON-TARGETplus SMARTpool siRNA (Thermo Scientific-Dharmacon), using 8 µl/well INTERFERin transfection reagent (Polyplus). Transfection overlay was carried out for 48 h, followed by an additional round of siRNA transfection identical to the first one. Eighteen h following the second siRNA transfection, the medium was replaced with serum-free and phenol-free DMEM for 72 h to reach serum starvation conditions. Following serum starvation, both scramble and MSK1siRNA or MSK2 siRNA knockdown cells were treated with 100 nM TPA for 0, 15, 30, 45 or 60 min. Following induction with TPA, the medium was removed and cells washed 2X with 1X PBS and harvested by scraping. Samples were micro-centrifuged at 2000 rpm for 10 min, and PBS supernatant removed by aspiration. Pellets were frozen at −80°C prior to processing for RNA extraction. For each trial, a set of scramble, MSK1 and MSK2 siRNA treated wells were set aside for protein extraction and analysis of protein knockdown by immunoblot using antibodies against MSK1 (Sigma, M5437), MSK2 (Invitrogen, 38-5300), or actin for a loading control (A5441).

### Immunofluoresence of MSK1 and MSK2

Indirect immunolocalization was performed as described previously [31]. Goat polyclonal antibodies against MSK1 (1∶100, Santa Cruz, sc-9392) and rabbit polyclonal antibodies against MSK2 (1∶100, Invitrogen, 38-5300) were used as the primary antibodies. Alexa Fluor 488 donkey anti-rabbit IgG (Invitrogen) and Alexa Fluor 568 donkey anti-goat IgG (Invitrogen) were used as the secondary antibodies. DNA was counterstained with 4′, 6-diamidino-2-phenylindole (DAPI). The coverslips were mounted onto glass slides using Prolong Gold anti-fade reagent (Invitrogen). Primary-antibody-omission control experiments demonstrated the specificity of the antibodies used. Digital images were captured with Zeiss Axio Imager Z1 microscope and AxioCam HRm camera. The stacked images were captured with 100 slices at stepwise of 200 nm. The deconvolution analysis of stacked images was done with the AxioVision software (Carl Zeiss).

### Alignment of Human MSK1 and MSK2 Amino Acid Sequences

MSK1, also known as RPS6KA5 (ribosomal protein S6 kinase alpha-5) and MSK2 or RPS6KA4 amino acid sequence entries #075582 and #075676 from the UniProtKB database, respectively were aligned using the freeware ClustalX (Fig. S1). Annotation was generated by the freeware GeneDoc.

## Results

### MSK1 and MSK2 are in Different Complexes

**Figure 1:**
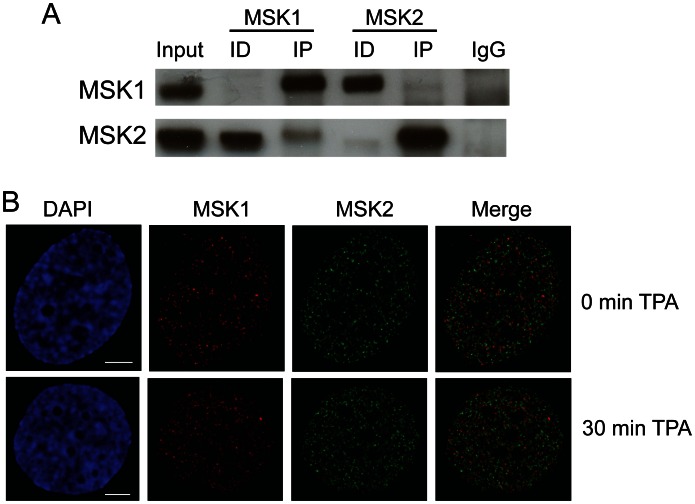
MSK1 and MSK2 are in different complexes. A. MCF-7 cell lysate (500 µg) was incubated with anti-MSK1 (rabbit, Sigma; 4 µg) or anti-MSK2 (rabbit, Invitrogen; 2 µg) antibodies. The immunoprecipitates (*IP*) and equivalent volumes of lysate (Input), immunodepleted (*ID*) fractions, and IgG control (*IgG)*, corresponding to 50 µg of lysate, were loaded onto SDS-8% polyacrylamide gels, transferred to nitrocellulose membranes, and immunochemically stained with anti-MSK1 or anti-MSK2 antibodies. B. MCF-7 cells grown on coverslips were serum starved and then treated with or without TPA as described in *Materials and Methods*. The cells were fixed and immunostained with antibodies against MSK1 and MSK2, and co-stained with DAPI. Spatial distribution was visualized by fluorescence microscopy and image deconvolution was done by AxioVision software. Yellow signal in the merged images indicates colocalization. Bar, 5 µm.

Immunoblot analyses of MCF-7 cell lysate show that both MSK1 and MSK2 are expressed in these breast cancer cells (Fig. 1A), in agreement with previously published immuno-histochemistry data [13]. To determine if MSK1 and MSK2 are found in the same protein complex, we performed immunoprecipitation assays using antibodies against each MSK isoform. Fig. 1A shows that following incubation of MCF-7 cell lysates with anti-MSK1 antibodies, MSK1 was recovered in the immunoprecipitated fraction as expected, while most of MSK2 was found in the immunodepleted fraction. Likewise, following incubation with anti-MSK2 antibodies, MSK1 was in immunodepleted fraction, while most MSK2 was in the immunoprecipitated fraction. Note that neither MSK1 nor MSK2 could be detected in the IgG negative control fraction (Fig. 1A). These results show that MSK1 and MSK2 have a very low level of association, that is the majority of MSK1 and MSK2 are not in the same multiprotein complex in MCF-7 cells. The differential localization of MSK1 and MSK2 in either IP or ID fractions also indicated that the antibodies against MSK1 and MSK2 were specific to each isoform (Fig. 1A).

MSK1 and MSK2 localization was assessed by immunofluoresence using antibodies specific to each kinase isoform (Fig. 1B). In either serum starved or TPA induced (30 min) conditions, MSK1 and MSK2 did not colocalize. To verify this finding, we also tested another ER+ breast cancer cell line, ZR75 for MSK1 and MSK2 localization and found that MSK1 and MSK2 also did not associate in these cells under serum starvation or TPA induction conditions (Fig. S2). Taken together, these data indicate that MSK1 and MSK2 have a low level of association by immunoprecipitation and do not colocalize in the cell, thereby suggesting that the majority of MSK1 and MSK2 exist in distinct complexes.

### Recruitment of MSK1 and MSK2 to *TFF1* Gene Regions in Response to ERK-MAPK Signaling

Here, we used the high-resolution chromatin immunoprecipitation (ChIP) assay to determine the MSK1, MSK2 and H3S10ph distribution along the regulatory and coding regions of the *TFF1* gene in response to ERK-MAPK signaling. The regions chosen for analysis are shown in Fig. 2A. Following stimulation of serum starved MCF-7 cells with TPA for 0, 15, 30, 45 and 60 min, nuclei from formaldehyde treated cells were isolated, and the chromatin was processed to mononucleosomal length with MNase [7]. Upon induction with TPA, the association and dissociation of MSK1 and MSK2 with the *TFF1* enhancer (−10476) and UPE (−429) followed similar time courses (Fig. 2B). Binding of both H3 kinases to the *TFF1* enhancer and UPE peaked at 30 min following TPA stimulation of MCF-7 cells. There was no TPA-induced association of MSK1 and MSK2 with the *TFF1* coding region (+2045 and +4147) (Fig. 2B). As MSK1 and MSK2 target the H3 N-terminal tails, we used antibodies against H3S10ph to immunoprecipitate formaldehyde-crosslinked mononucleosomes from quiescent MCF-7 cells treated with TPA. The positioning of H3S10ph at the regulatory and coding regions of *TFF1* mirrors that of MSK1 and MSK2; H3S10ph levels are present and inducible only at the regulatory regions and not at the coding region of *TFF1* (Fig. 2B).

### Association of 14-3-3 Proteins with *TFF1* Regulatory Regions in Response to ERK-MAPK Signaling

Several isoforms of 14-3-3 proteins have been identified as “readers” of H3S10ph signatures at the chromatin of inducible genes [33]. The binding affinity of 14-3-3 isoforms for H3S10ph has been reported to be more stable if the K14 residue is acetylated [34], [35]. H3S10phK14ac is also associated with transcriptional activation [6]. With this in mind, we performed ChIP assays with antibodies against H3S10phK14ac to determine the distribution of this dual H3 modification along the *TFF1* gene. Fig. 2C shows that TPA rapidly increased H3S10phK14ac levels at the enhancer and UPE regulatory regions, but not the coding region of *TFF1*. Since 14-3-3 isoforms are downstream effectors of H3S10phK14ac [33], we immunoprecipitated mononucleosomal chromatin from formaldehyde-crosslinked TPA-stimulated MCF-7 cells with antibodies against 14-3-3ε and 14-3-3ζ. Upon TPA induction, recruitment of 14-3-3ε/ζ isoforms mirrored the positioning of H3S10phK14ac at both 5′ regulatory regions of *TFF1*, but not at the coding region (Fig. 2C), suggesting that MSK mediates H3 modifications and the subsequent recruitment of proteins that recognize those nucleosomal chromatin signatures at the regulatory regions of inducible genes.

### ERK-MAPK-induced Recruitment of BAF/PBAF Chromatin Remodeling Complex and RNA Polymerase II at *TFF1* Gene Regions

Chromatin of inducible genes requires not only histone modifications, but also simultaneous rapid remodeling in order to allow access of transcription factors and RNA polymerase II (RNAPII) to specific DNA sequences [36]. BAF/PBAF chromatin remodeling complexes (human homologs of SWI/SNF, [37]) have been implicated in localized remodeling of nucleosomes to allow binding of various factors which aid in the initiation of transcription [30]. Positioning of chromatin remodelers is polarized towards the 5′ end of inducible genes [38], [39]. However, distribution of chromatin remodelers along *TFF1* gene in response to ERK-MAPK signaling has not been studied. We performed the high resolution ChIP assay with antibodies against BRG1, the ATPase subunit of BAF/PBAF remodeling complex, in TPA-treated MCF-7 cells. Fig. 2D shows that TPA stimulation of MCF-7 cells increased the loading of BRG1 at the enhancer and UPE regulatory regions of *TFF1*, while there was no binding of BRG1 to the coding region. Presence of BRG1 at the promoter region of *TFF1* suggests that the local chromatin might be favorable for RNAPII binding. Therefore, we performed ChIP assays with an antibody against the initiation-engaged form of RNAPII that is phosphorylated at serine 5 (RNAPII S5ph) or an antibody that does not differentiate between phosphorylated and non-phosphorylated forms of RNAPII (total RNAPII). Fig. 2D shows that TPA treatment of MCF-7 cells increased the levels of RNAPII S5ph and RNAPII at the promoter region of *TFF1*. Binding of either RNAPII S5ph or RNAPII was not detected at the enhancer region. Furthermore, RNAPII antibody, which recognizes all forms of RNAPII, detected inducible positioning of RNAPII along the coding region of *TFF1* (Fig. 2D).

### TPA-induced MSK1 or MSK2 Co-occupancy with H3S10ph at *TFF1* Regulatory Regions

**Figure 3:**
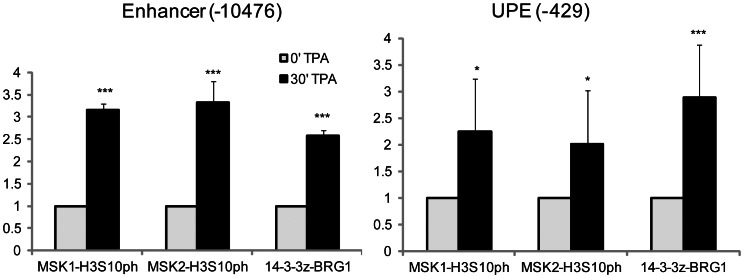
TPA-induced co-occupancy of MSK1/H3S10ph, MSK2/H3S10ph and 14-3-3ζ/BRG1 at regulatory regions of *TFF1*. Re-ChIP experiments were performed on formaldehyde-crosslinked mononucleosomes prepared from serum-starved MCF-7 cells either untreated or treated with TPA for 30 min. The antibodies were used as indicated in the graph. Equal amounts of input and immunoprecipitated DNA were quantified by real-time quantitative PCR. The enrichment values of the *TFF1* enhancer (-10476) and UPE (-429) sequences are the mean of three independent experiments, and the error bars represent the standard deviation. *P≤0.05, **P≤0.01, ***P≤0.001 (Student’s paired t-test).

To determine whether MSK1 co-occupies the enhancer and UPE with H3S10ph, we performed sequential ChIP assays (re-ChIP) with mononucleosomes prepared from TPA- treated and formaldehyde-cross linked MCF-7 cell lysates. Fig. 3 demonstrates that MSK1 is simultaneously present with H3S10ph at enhancer and UPE regions of *TFF1* gene after 30 min of TPA stimulation. Similarly, MSK2 can also be found with H3S10ph at both the enhancer and UPE of *TFF1* (Fig. 3). These results indicate that the MSK1 or MSK2 present at the regulatory regions of *TFF1* are catalytically active and therefore mediate phosphorylation of H3S10 at these regions. The phosphoserine recognizing 14-3-3 proteins can bind to H3S10ph [33] and may recruit other proteins, such as components of the BAF/PBAF remodeling complex to aid in transcriptional activation [7]. Here we performed re-ChIP assays with anti-14-3-3ζ and BRG1 antibodies at the enhancer and UPE region of *TFF1* (Fig. 3). TPA was able to induce co-occupancy of 14-3-3ζ and BRG1 at the enhancer and UPE regions of *TFF1*. Together these results show that either MSK1 or MSK2 recruited to the *TFF1* UPE and enhancer phopshorylates H3 at S10, leading to the binding of 14-3-3ε/ζ and recruitment of BRG1.

### H89 Inhibition of MSK-mediated Nucleosomal Response at the Regulatory Regions of the *TFF1* Gene

**Figure 4:**
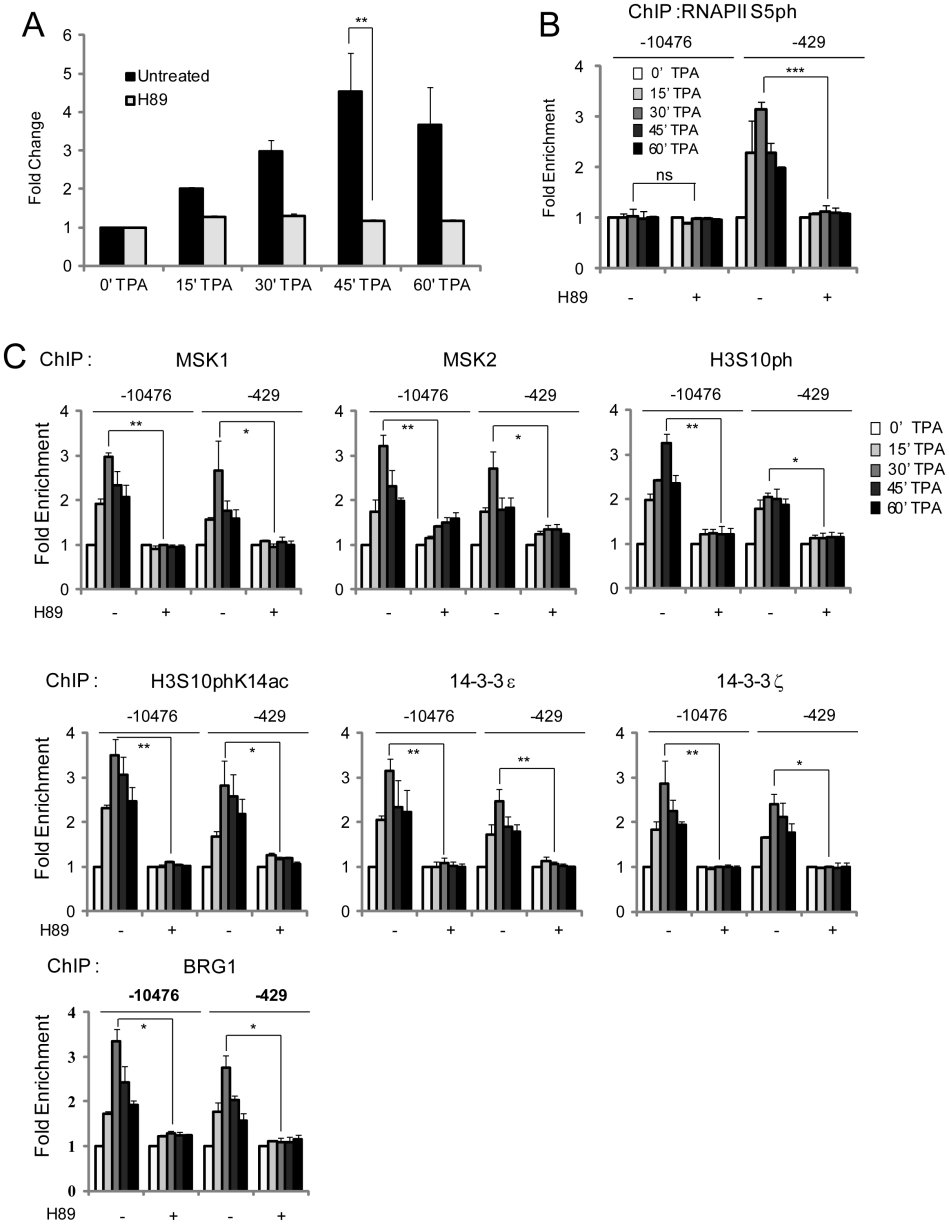
Effect of H89 on TPA-induced expression, nucleosomal response and chromatin remodeling of *TFF1*. Serum-starved MCF-7 cells were pre-treated or not with H89 prior to TPA stimulation for 15, 30, 45 and 60 min. A. Total RNA was isolated and quantified by real time RT-PCR. Fold change values, normalized to *CYP33* expression levels and time 0 values, are the mean of three independent experiments, and the error bars represent the standard deviation. B. Formaldehyde-crosslinked mononucleosomes were prepared and used in ChIP assays with antibodies against RNAPII S5ph. Equal amounts of input and immunoprecipitated DNAs were quantified by real-time quantitative PCR. The enrichment values of the *TFF1* enhancer (−10476) and UPE (−429) sequences are the mean of three independent experiments, and the error bars represent the standard deviation. C. Serum-starved MCF-7 cells were pre-treated or not with H89 prior to TPA stimulation for 15, 30, 45 and 60 min. Formaldehyde-crosslinked mononucleosomes were prepared and used in ChIP assays with antibodies against MSK1, MSK2, H3S10ph, H3S10phK14ac, 14-3-3ε, 14-3-3ζ, and BRG1. Equal amounts of input and immunoprecipitated DNAs were quantified by real-time quantitative PCR. The enrichment values of the *TFF1* enhancer (-10476) and UPE (-429) sequences are the mean of three independent experiments, and the error bars represent the standard deviation. *P≤0.05, **P≤0.01, ***P≤0.001, ns = P≥0.05 (Student’s paired t-test).

At a concentration of 10 µM, the MSK inhibitor H89 has been shown to selectively inhibit the nucleosomal response [8]. To validate the role of the MSK-mediated nucleosomal response in the TPA-induced transcription of *TFF1*, we performed real time RT-PCR on cDNA isolated from TPA-treated MCF-7 cells that were previously exposed or not to H89. Fig. 4A shows that TPA induced the expression of *TFF1*, with the expression peaking at 45 min after TPA stimulation. Presence of H89 decreased *TFF1* expression in response to TPA (Fig. 4A). Conversely, H89 did not affect the expression of cyclophilin 33 (*CYP33*) (data not shown). To determine if *TFF1* transcription was inhibited by H89 at the initiation level, we analyzed the effect of H89 on the occupancy of the initiation-engaged form of RNAPII (RNAPII S5ph) at the regulatory regions. Fig. 4B shows that RNAPII S5ph was not present at the *TFF1* promoter following H89 treatment. These results suggest that MSKs mediate chromatin-remodeling events resulting in the initiation of *TFF1* transcription. In support of this implication, we found that TPA-induced expression of *TFF1* was abolished by the ERK1/2 inhibitor UO126, but was not affected by the PKA inhibitor RpcAMP (data not shown).

Since MSKs mediate the nucleosomal response, we wanted to examine the events occurring at the regulatory regions of *TFF1* in the presence of H89. We exposed quiescent MCF-7 cells with H89 for 30 min, prior to TPA stimulation and performed ChIP assays. Fig. 4C shows that H89 pre-treatment of MCF-7 cells markedly decreased TPA-induced recruitment of MSK1 and MSK2 at the enhancer and UPE regulatory regions of *TFF1*. The levels of H3S10ph and H3S10phK14ac were also dramatically reduced in the presence of H89. Similarly, TPA-induced binding of phospho-H3 effectors, 14-3-3ε and 14-3-3ξ was abolished at the enhancer and UPE regions of *TFF1*. Prior exposure of MCF-7 cells to H89 prevented TPA-induced association of BRG1 with both regulatory regions of *TFF1* (Fig. 4C).

### Attenuated TPA-induced Expression of *TFF1* in MSK1 or MSK2 Knockdown MCF-7 Cells

**Figure 5:**
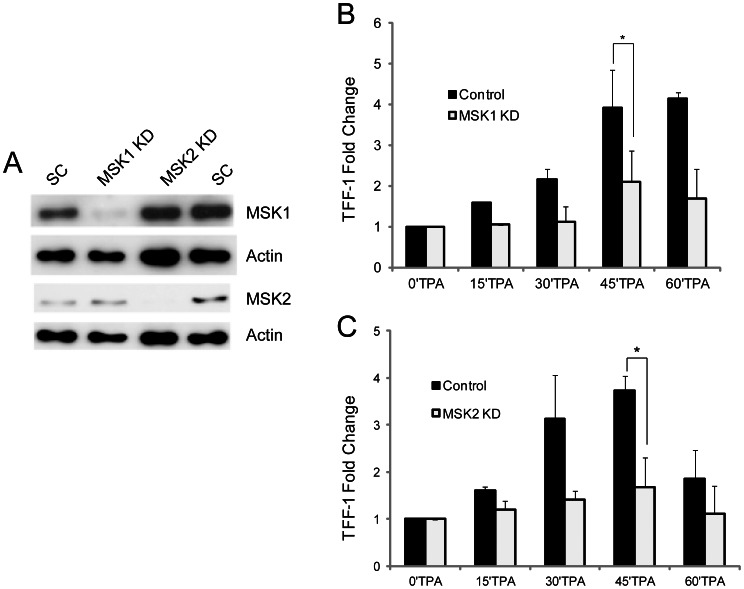
MSK1 and MSK2 activity contribution to TPA-induced *TFF1* expression. A. Levels of MSK1 and MSK2 in transient MSK1 or MSK2 knockdown and scramble control (SC) MCF-7 cells were analyzed by immunoblotting. Actin was used as loading control. Note that MSK1 (7 µg, rabbit, Sigma) antibody was used at a higher titer than MSK2 (2.5 µg, rabbit, Invitrogen). B, C. Serum-starved transient MSK1 or MSK2 knockdown and scramble control MCF-7 cells were treated with TPA for 0, 15, 30, 45 and 60 min. Total RNA was isolated and quantified by real time RT-PCR. Fold change values, normalized to *GAPDH* expression levels and time 0 values, are the mean of three independent experiments, and the error bars represent the standard deviation. *P≤0.05 (Student’s paired t-test).

Since H89 inhibits the activities of MSK1 and MSK2, we individually knocked down MSK1 and MSK2 to evaluate the contribution of each enzyme to the TPA-induced expression of *TFF1*. MCF-7 cells were transiently transfected with MSK1 siRNA, MSK2 siRNA or scramble (non-targeting) siRNA. Levels of residual MSK1 and MSK2 protein in MSK1 or MSK2 knockdown and control MCF-7 cells were determined by immunoblot analyses (Fig. 5A). The average knockdown values in three separate experiments were 66±14% (81% for the experiment shown in Fig 5A) for MSK1 and 93±1% for MSK2. MSK2 levels were unaltered in MSK1 knockdown MCF-7 cells, and conversely, MSK1 levels were not affected by MSK2 knockdown. MSK1 or MSK2 knockdown and control MCF-7 cells were serum-starved and stimulated with TPA for various periods of time. The graph in Fig. 5B shows the average TPA-induced expression of *TFF1* in MSK1 knockdown compared to control MCF-7 cells, from three separate experiments. MSK1 knockdown resulted in a 50% reduction of *TFF1* expression. TPA-induced expression of *TFF1* in MSK2 knockdown was similarly decreased compared to control MCF-7 cells. (Fig.5C). These results indicate that both MSK1 and MSK2 contribute to the TPA-induced expression of *TFF1* in MCF-7 cells. We have recapitulated these results using ZR75 cells in which TFF1 expression was markedly reduced by MSK1 or MSK2 siRNA transient knockdown (Fig. S3).

### Knockdown of MSK1 or MSK2 does not Change AP1 Protein Levels

**Figure 6:**
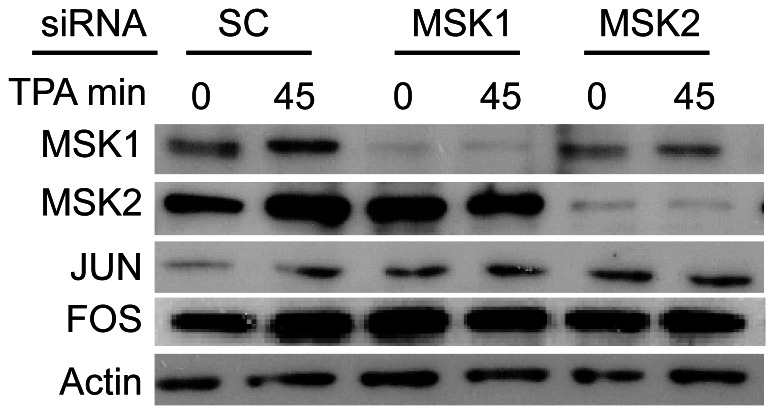
FOS and JUN levels do not change with MSK1 or MSK2 knockdown in MCF-7 cells. MCF-7 cells were treated with scramble, MSK1, or MSK2 siRNA, serum starved for 72 h, and treated with TPA for 0 or 45 min. Protein lysates were analyzed by immunoblotting using antibodies against MSK1 (rabbit, Sigma), MSK2 (rabbit, Abcam ab99411), JUN, or FOS. Actin was used as a loading control.

We show that MSK1 and MSK2 are recruited to the *TFF1* enhancer and UPE regions in response to TPA. The *TFF1* UPE contains DNA binding elements for transcription factors, including the activator protein 1 (AP1). AP1 constitutes a combination of dimers formed by members of the JUN, FOS and ATF families of transcription factors. We have previously shown that mutation of the AP1 binding site abrogated the TPA-induced transcriptional response of a reporter gene under the control of the *TFF1* promoter, demonstrating the requirement for the AP1 site [27]. We also showed that JUN was recruited to the *TFF1* UPE in response to TPA [27]. Furthermore, in mouse fibroblast cells, JUN was found to be part of the MSK1 complex (unpublished data). These observations imply that the recruitment of MSKs to *TFF1* UPE is mediated by transcription factors like JUN. We have previously reported that in Hras1-transformed mouse fibroblasts, the transcription of AP1 family members JUN and FRA-1 are affected by MSK inhibition and that knockdown of MSK1 leads to a reduction of JUN and FRA-1 protein levels [5]. AP1 is required for its own expression, as well as *TFF1* expression [24], [40]. To determine whether knockdown of MSK1 or MSK2 changed the levels of AP1 members, we performed an immunoblot analyses of JUN and FOS from cell lysates of control and MSK knockdown cells. Fig. 6 shows that neither MSK1 nor MSK2 knockdown altered the levels of JUN and FOS. These observations provide evidence that the attenuated TPA-induced expression of the *TFF1* gene in MSK1 or MSK2 knockdown cells is not a consequence of reduced AP1.

## Discussion

Our studies demonstrate that MSK1 and MSK2 contribute to the TPA-induced expression of *TFF1* in MCF-7 cells. Moreover, we show for the first time that MSK1 and MSK2 are involved in nucleosome remodeling at an enhancer.

MSK1 and MSK2 are often both expressed in mammalian cells, and there is evidence that these two kinases have different functions. Adding to this knowledge, we show that MSK1 and MSK2 are in distinct nuclear locations and in separate complexes in breast cancer cells. However, either kinase can associate with the *TFF1* enhancer and promoter regions and phosphorylate H3 at S10. As with immediate-early genes in mouse fibroblasts, the MSK-induced H3S10ph and H3S10phK14ac are limited to the regulatory regions of the *TFF1* gene and are not found within the gene body. Most likely, transcription factors such as AP1 recruit MSK1 or MSK2 and anchor these enzymes such that only the H3 in nucleosomes in the immediate vicinity of the kinases will be modified. Such restrictions in MSK activity then localize the recruitment of 14-3-3ε/ζ and the BAF/PBAF remodeling complex, resulting in localized chromatin remodeling events which support further transcription factor binding and formation of a productive pre-initiation complex [7].

The requirement of both MSK1 and MSK2 in the TPA-induction of the *TFF1* gene in human breast cancer cells is clearly shown in studies with the MSK inhibitor H89 and in the MSK1 and MSK2 knockdown cells. There are several possible explanations for the requirement of both enzymes for maximal induction of the *TFF1* gene. First, MSK1 and MSK2 levels could be limiting, such that both are required for maximal TPA-induced expression of *TFF1*. Secondly, MSK1 and MSK2 might be recruited to *TFF1* UPE and enhancer by different transcription factors (such as different AP1 complexes). Thirdly MSK1 and MSK2 may participate in the activation of each other. The activation of MSK1 and MSK2 is a multi-step process that has not been completely elucidated [41], [42]. The MSK1 activation model postulates that after being phosphorylated by active ERK1/2 or p38 MAPK, MSK1 C-terminal kinase domain phosphorylates Ser 212 in the N-terminal kinase domain and Ser 376 and Ser 381 in the linker region between the two domains. Once phosphorylated, MSK1 N-terminal domain is able to phosphorylate substrates as well as three sites at the C-terminus. Perhaps, MSK2 is involved in the phosphorylation of MSK1 Ser 212, Ser 376 and Ser 381 residues. Reciprocally, MSK1 might contribute to the phosphorylation of MSK2 corresponding Ser residues. Regardless of the mechanism(s), our study shows that MSK1 and MSK2 are both required for full TPA-induction of the immediate early gene *TFF1*.

TFF1 protein is known to enhance the migration, invasion, and metastatic potential of breast cancer cells both *in vitro* and *in vivo* [19], [43]. A recent study has reported that the mechanism of increased breast cancer migration/invasiveness involves MAPK-induced MSK1 recruitment to the Snail promoter, via activation of the CXCL5-CXCR2 chemotaxis ligand-receptor pathway [44]. The induction of Snail leads to transcriptional repression of E-cadherin and the induction of epithelial-mesenchymal transition and cell migration [45], [46]. Interestingly, the forced expression of *TFF1* in DU145 prostate cancer cells led to increased expression of Snail as well as decreased expression of E-cadherin [47]. Additionally, MSK1-mediated Snail production in breast cancer cells may lead to increased metastasis of breast cancer cells to bone tissue [44]. Therefore, the mechanism of MSK-mediated *TFF1* gene transcription in breast cancer may hold important insights into breast cancer progression and merits further study.

## Supporting Information

Figure S1 **Alignment of MSK1 and MSK2 amino acid sequences.** Identical amino acids are shaded in black. Numbering above the sequence indicates position in MSK1, and numbering on the right side indicates position in MSK1 or MSK2. The blue bar represents the epitope recognized by the MSK1 antibody used in these studies, while the red bar represents the epitope region recognized by the MSK2 antibody.
(TIF)

Figure S2 **MSK1 and MSK2 do not colocalize in ZR75 cells.** ZR75 cells grown on coverslips were serum starved and then treated with or without TPA as described in *Materials and Methods*. The cells were fixed and immunostained with antibodies against MSK1 and MSK2, and co-stained with DAPI. Spatial distribution was visualized by fluorescence microscopy and image deconvolution was done by AxioVision software. Yellow signal in the merged images indicates colocalization. Bar, 5 µm.
(TIF)

Figure S3 **MSK1 and MSK2 activity contribute to TPA-induced** ***TFF-1*** **expression in ZR75 cells.** A. Serum-starved transient MSK1 or MSK2 knockdown and scramble control ZR75 cells were treated with TPA for 0 or 45 min. Total RNA was isolated and quantified by real time RT-PCR. Fold change values, normalized to *GAPDH* expression levels and time 0 values, are the mean of three independent experiments, and the error bars represent the standard deviation. B. Quantification of MSK1 protein levels in scramble treated or MSK1 siRNA treated cells in A. MSK1 levels were normalized to β-actin levels and are the mean of three independent experiments, where the error bars represent the standard deviation. Protein expression values are expressed as Integrated Density Values (*IDV*) C. Quantification of MSK2 protein levels in scramble treated or MSK2 siRNA treated cells in A. MSK2 levels were normalized to β-actin and are the mean of three independent experiments, where error bars represent the standard deviation. *P≤0.05 (Student’s paired t-test).
(TIF)
