# Supplementary figures and images for: Mitogen- and Stress-Activated Protein Kinases 1 and 2 Are Required for Maximal Trefoil Factor 1 Induction
Source: PLoS One. 2013 May 13;8(5):e63189. doi: 10.1371/journal.pone.0063189 (PMC3652853; doi:10.1371/journal.pone.0063189)

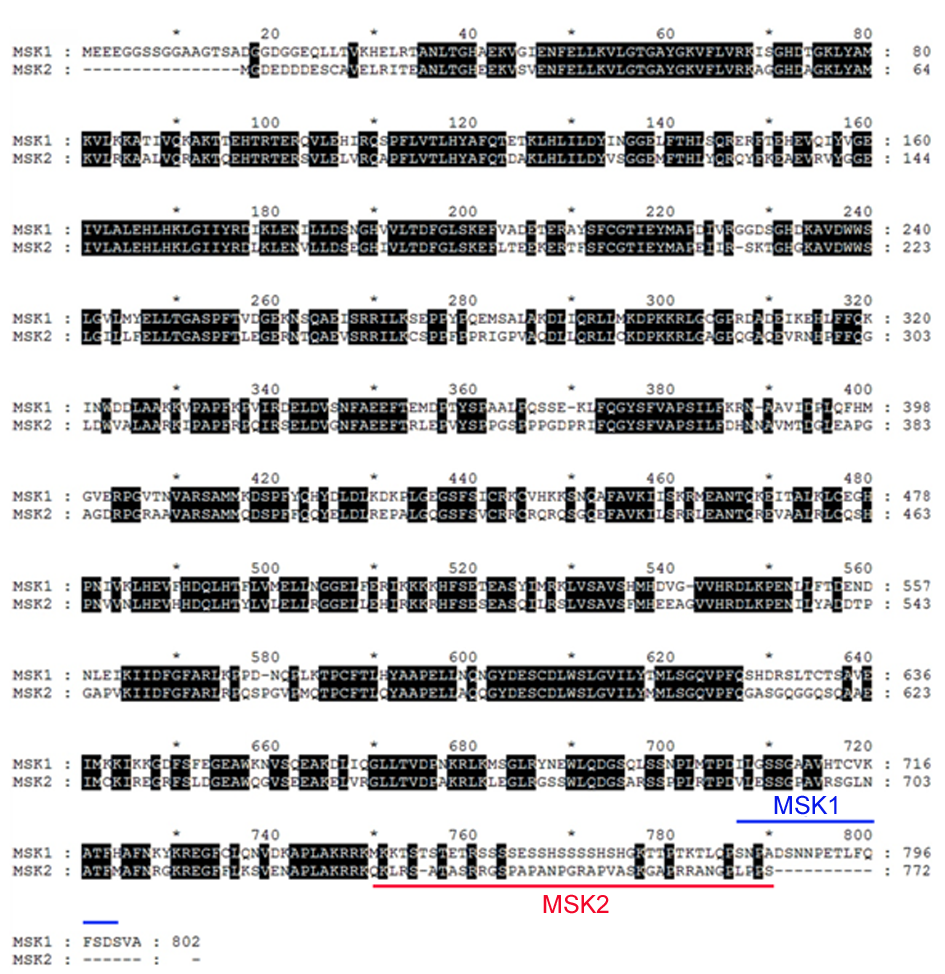

Supplement: Figure S1 — Alignment of MSK1 and MSK2 amino acid sequences. Identical amino acids are shaded in black. Numbering above the sequence indicates position in MSK1, and numbering on the right side indicates position in MSK1 or MSK2. The blue bar represents the epitope recognized by the MSK1 antibody used in these studies, while the red bar represents the epitope region recognized by the MSK2 antibody. (TIF) [file pone.0063189.s001.tif]

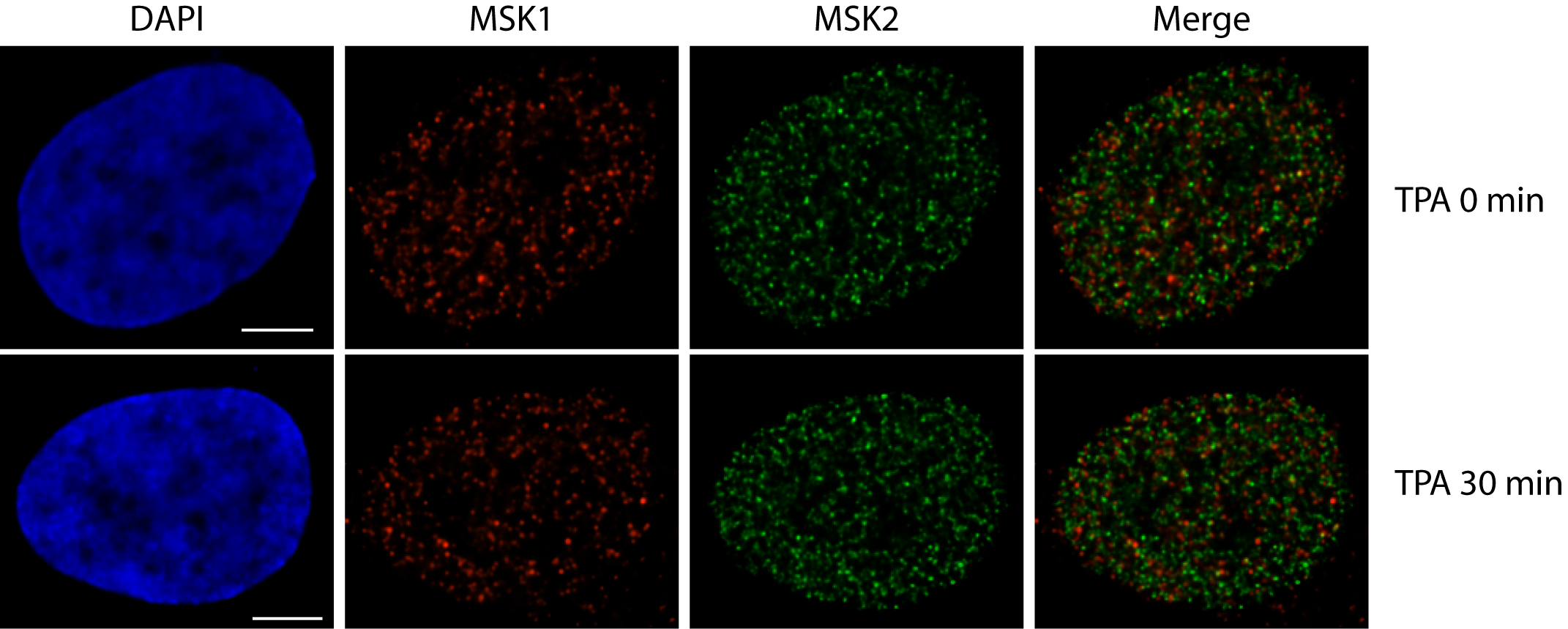

Supplement: Figure S2 — MSK1 and MSK2 do not colocalize in ZR75 cells. ZR75 cells grown on coverslips were serum starved and then treated with or without TPA as described in Materials and Methods . The cells were fixed and immunostained with antibodies against MSK1 and MSK2, and co-stained with DAPI. Spatial distribution was visualized by fluorescence microscopy and image deconvolution was done by AxioVision software. Yellow signal in the merged images indicates colocalization. Bar, 5 µm. (TIF) [file pone.0063189.s002.tif]

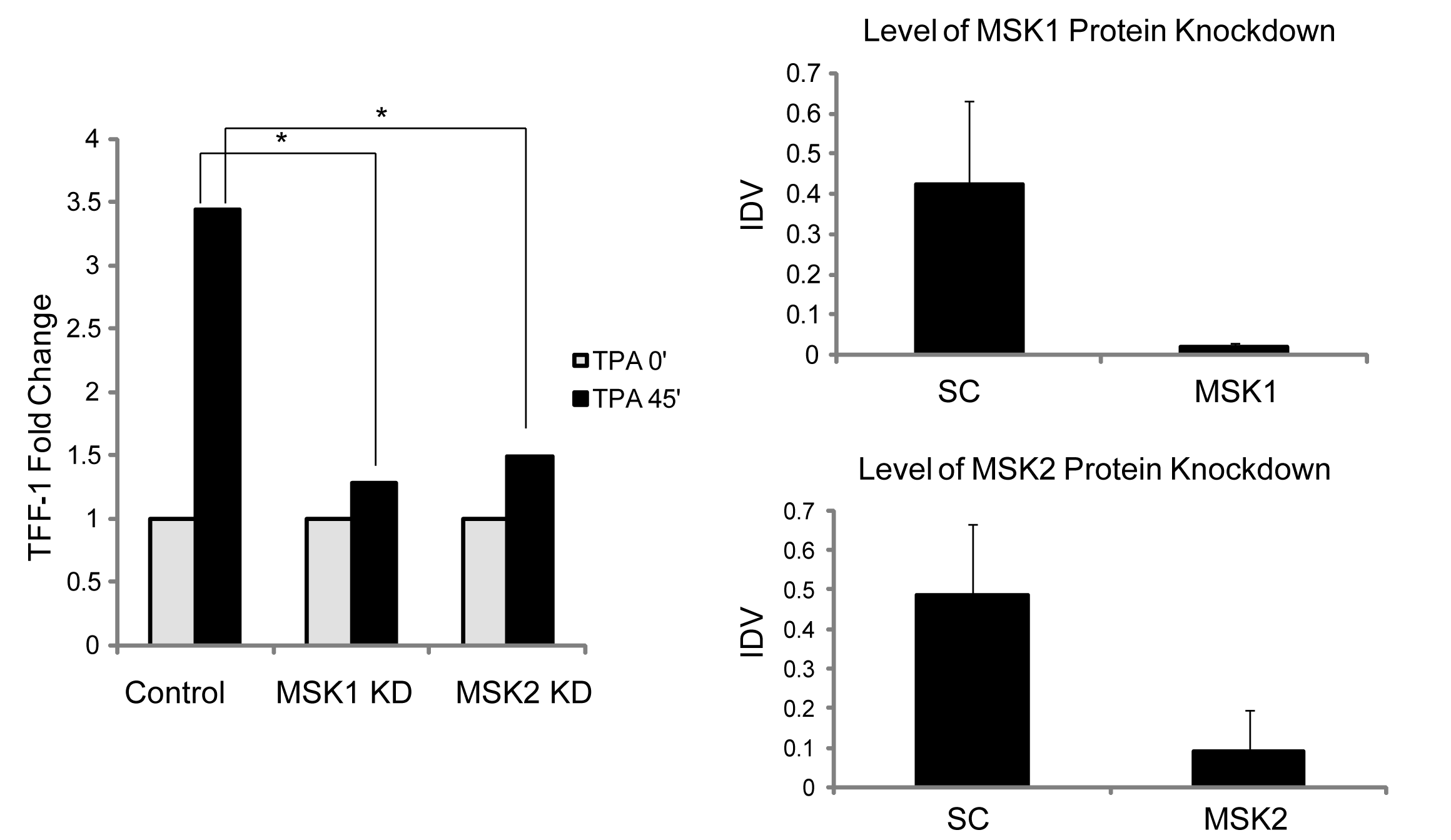

Supplement: Figure S3 — MSK1 and MSK2 activity contribute to TPA-induced TFF-1 expression in ZR75 cells. A. Serum-starved transient MSK1 or MSK2 knockdown and scramble control ZR75 cells were treated with TPA for 0 or 45 min. Total RNA was isolated and quantified by real time RT-PCR. Fold change values, normalized to GAPDH expression levels and time 0 values, are the mean of three independent experiments, and the error bars represent the standard deviation. B. Quantification of MSK1 protein levels in scramble treated or MSK1 siRNA treated cells in A. MSK1 levels were normalized to β-actin levels and are the mean of three independent experiments, where the error bars represent the standard deviation. Protein expression values are expressed as Integrated Density Values (IDV) C. Quantification of MSK2 protein levels in scramble treated or MSK2 siRNA treated cells in A. MSK2 levels were normalized to β-actin and are the mean of three independent experiments, where error bars represent the standard deviation. *P≤0.05 (Student’s paired t-test). (TIF) [file pone.0063189.s003.tif]
